# The Relationship Between Body Dysmorphic Symptoms, Depressive Symptoms and Suicidality – A Mediation Analysis

**DOI:** 10.32872/cpe.17121

**Published:** 2026-02-27

**Authors:** Hannah Vogel, Jens Barenbrügge, Julia Jenisch, Johanna Schulte, Ulrike Buhlmann

**Affiliations:** 1 Department of Psychology and Sports Sciences, University of Münster, Münster, Germany; University of Mannheim, Mannheim, Germany

**Keywords:** body dysmorphic disorder, suicidality, depression, mediation

## Abstract

**Background:**

Body dysmorphic disorder (BDD) is characterized by an excessive preoccupation with perceived appearance flaws, often accompanied by repetitive behaviors such as frequent mirror checking. It is further associated with severe occupational and social impairments, depression, and high levels of suicidality, including suicidal thoughts, suicide attempts and completed suicide. Given the high rates of depression and suicidality found in BDD, this study aimed to examine whether BDD symptoms may also be directly linked to suicidality, independent of depressive symptoms.

**Method:**

A mediation analysis was conducted to investigate the relationships between BDD symptoms, depression, and suicidality. Cross-sectional data were collected through an online self-test for BDD, assessing BDD symptoms, depression and suicidality. A total of 1,256 participants (aged 18–71; 72% female) met DSM-5 criteria for BDD (based on self-report).

**Results:**

Depressive symptoms partially mediated the relationship between BDD symptoms and suicidality. However, a direct association between BDD symptoms and suicidality was also observed, indicating that suicidality in individuals with BDD is not solely attributable to comorbid depressive symptoms.

**Conclusion:**

The findings underscore the need for thorough suicidality assessments in individuals with BDD, regardless of the presence of depressive symptoms. This further highlights the importance of targeted interventions to address suicidality in this population.

## Highlights

- BDD is associated with high suicidality.
- More severe BDD symptoms predict higher levels of depression.
- Depressive symptoms can only partly explain the relationship between BDD symptoms and suicidality.
- BDD symptoms directly contribute to increased suicidality after controlling for depressive symptoms.

Body dysmorphic disorder (BDD) is characterized by an excessive preoccupation with perceived physical defects that are not (or only slightly) visible to others (e.g., facial flaws regarding the size or shape of the nose or eyes; American Psychiatric Association [APA], 2013). The appearance concerns often trigger intense negative emotions such as guilt, shame, and anxiety (e.g., Phillips, 2014). Engaging in repetitive actions, both mentally and observably, is also common in BDD, and the disorder is associated with significant impairments, ranging from avoidance of social interactions, and difficulty concentrating at work to the inability to maintain relationships, work, or even leave the house (Phillips et al., 2007a). The point prevalence of BDD is estimated to be around 2%, surpassing the rates of disorders like schizophrenia and anorexia nervosa (APA, 2013). Surprisingly, despite its prevalence and impact, BDD is still often under-recognized in clinical settings (Veale et al., 2016).

## Associations Between BDD, Depression and Suicidality

Individuals with BDD face elevated risks of suicidal ideation and attempts, as outlined in a meta-analysis by Angelakis et al. (2016). Moreover, BDD often coexists with major depressive disorder (MDD; Gunstad & Phillips, 2003), which is itself strongly associated with suicidality (Brown et al., 2000; Cai et al., 2021; Dong et al., 2018). While MDD is the most common comorbidity of BDD (Phillips et al., 2007a), most individuals with MDD do not concurrently experience BDD (Brawman-Mintzer et al., 1995). Previous findings suggest that BDD and comorbid major depression are associated with increased suicidality (e.g., Phillips et al., 2005c). Specifically, in their longitudinal study of 200 individuals with BDD, lifetime suicidal ideation, suicide attempts, comorbidities and functional impairment were assessed using structured interviews. Logistic regression analyses showed high lifetime rates of suicidal ideation (78%) and attempts (27.5%). Functional impairment due to BDD predicted suicidal ideation and attempts. Comorbid MDD was linked specifically to suicidal ideation. These results suggest that multiple factors, including MDD, contribute to suicidality in BDD. However, a mediation model was not tested in the study by Phillips et al. (2005c). Shaw et al. (2016) investigated potential mediators of the relationship between BDD symptoms and suicidality based on Joiner’s (2005) Interpersonal Theory of Suicide (IPTS). According to IPTS, suicidal desire emerges primarily from two psychological states: *thwarted belongingness,* the feeling of not being meaningfully connected to others and *perceived burdensomeness,* the belief that one is a burden to others. In their cross-sectional mediation model, Shaw and colleagues (2016) found that the association between BDD symptom severity and both key constructs was fully mediated by depressive symptoms. These findings imply that BDD symptoms may contribute to suicidality indirectly via increased depressive symptomatology, which in turn amplifies feelings of social disconnection and burden. In other words, based on their results, no direct link between BDD symptoms and suicidality would be expected once depression is accounted for. In contrast to this finding, several studies found a significant association between BDD symptoms and suicidality even after controlling for depressive symptoms, suggesting that BDD symptoms may independently contribute to heightened suicidality (Krebs et al., 2022; Snorrason et al., 2019).

## Research Relevance and Significance of the Project

While previous research has provided initial insights into the relationship between BDD, depressive symptoms and suicidality, findings remain inconsistent. Phillips et al. (2005c) identified MDD and functional impairment as significant independent predictors of suicidal ideation in BDD. Notably, their design and analyses did not assess mediation directly, leaving it unclear whether depressive symptoms explain the association between BDD severity and suicidality. Extending this line of research, Shaw et al. (2016) found evidence that the association between BDD symptoms and thwarted belongingness and perceived burdensomeness was fully mediated by depressive symptoms, suggesting an indirect pathway from BDD severity via depression to suicidal ideation. However, suicidality was not assessed directly in their study, limiting the conclusions regarding actual suicidal thoughts or behaviors. In contrast, other studies (e.g., Krebs et al., 2022; Snorrason et al., 2019) demonstrated that the association between BDD and suicidality persists even after controlling for depressive symptoms, pointing toward the existence of a direct effect in addition to any potential mediation through depression. These methodological and conceptual differences across studies likely contributed to the varying effects observed, highlighting the need for further research to clarify the interplay between BDD, depression, and suicidality. Thus, the present study seeks to clarify whether the association between BDD symptoms and suicidality is fully or only partially mediated by depressive symptoms. To address this, the total effect of BDD symptoms on suicidality will be decomposed into its direct and indirect components (via depressive symptoms), allowing for a more precise estimation of the magnitude of each effect. In contrast to Shaw et al. (2016), who examined risk factors for suicidality (thwarted belongingness and perceived burdensomeness), the aim of our study was to investigate suicidality, including suicidal thoughts and attempts. This deliberate distinction is essential for gaining a clearer understanding of the relationships between BDD symptomatology, depressive symptoms, and suicidality. Furthermore, these findings may hold clinical relevance by informing whether depressive symptoms should be a primary treatment target in reducing suicide risk in individuals with BDD — or whether BDD symptomatology itself constitutes an independent risk factor that requires direct therapeutic attention. To further explore the relationship between the three variables the following hypotheses are formulated:

H1: Depressive symptoms mediate the positive association between BDD symptoms and suicidality.

H2: There is a direct association between BDD symptoms and suicidality independent of depressive symptoms.

## Method

The cross-sectional study utilized an anonymous online survey format administered through Unipark (Questback GmbH, 2017), with a self-test for appearance concerns available online via the BDD Outpatient Clinic at the University of Münster (see Schulte et al., 2020). The study was approved by the responsible Ethics Committee at the University of Münster: 2017-32-JSch. Data and analysis code are available at the Open Science Framework (OSF; https://doi.org/10.17605/OSF.IO/AB8X4). Participants received instructions emphasizing voluntary participation, anonymity, and privacy. Informed consent was obtained both before and after data entry. Moreover, individualized feedback was provided after the completion of the survey, offering insights into potential BDD signs, comorbid diagnoses, and information on support services, including a telephone counseling service for individuals reporting suicidal ideation. The average completion time was 18 minutes (*SD* = 13 minutes).

### Sample

Between February 2016 and May 2018, and from March 2019 to June 2021, a total of 15,273 individuals accessed the self-test, with 3,452 completing it. Exclusions were made for individuals who did not consent to data use (*n* = 808), participated repeatedly (*n* = 89), lacked sufficient language skills (*n* = 5), were under 18 years old (*n* = 78), or did not meet the diagnostic criteria for BDD (*n* = 1,216) according to the self-report measure BDD-5 (Möllmann & Buhlmann, 2024), which is based on DSM-5 criteria (APA, 2013). To differentiate BDD from eating disorder (ED) symptoms, participants completed the SCOFF questionnaire (Morgan et al., 1999). It consists of five yes/no items assessing disordered eating behaviors (e.g., “Do you worry you have lost control over how much you eat?”). A cut-off of two or more affirmative responses has shown good diagnostic accuracy, with pooled sensitivity of .80 and specificity of .93 (Botella et al., 2013). Participants who scored above this cut-off were excluded from the analyses. Consequently, the final sample size was *n* = 1,256, ranging from 18 to 71 years of age (*M* = 30.1, *SD* = 9.9). The majority of participants (72.3%, *n* = 908) were female (see Table 1). Participants’ appearance concerns referred to: Skin (55.6%, *n* = 698), nose (48.3%, *n* = 607), hair (41.9%, *n* = 526), breast (31.0%, *n* = 389), mouth (23.6%, *n* = 296), genitals (22.2%, *n* = 279), eyes (22.1%, *n* = 278), hands (12.9%, *n* = 162), muscles (12.3%, *n* = 155), ears (8.3%, *n* = 104), body characteristics related to ethnicity (3.0%, *n* = 38), and other (18.5%, *n* = 232). The five most prevalent diagnoses reported, based on prior reported clinical professional assessments, were depressive disorders (43.6%, *n* = 547), social anxiety disorder (17.4%, *n* = 219), BDD (12.4%, *n* = 156), generalized anxiety disorder (10.8%, *n* = 136), and ED (11.9%, *n* = 149). Nearly two-thirds (63.6%, *n* = 799) reported having received at least one diagnosis of a mental disorder.

### Instruments/Measures

Demographic data were collected on the variables age, gender, place of residence (country), highest educational degree, current occupation, and marital status (see Table 1).

**Table 1:** Demographic Data

Demographic Variable	%	*n*
Country		
Germany	88.5	1112
Austria	4.9	62
Switzerland	5.4	68
Other	1.1	14
Highest Educational Degree		
None	0.9	11
10 Years of School	26.7	335
11-13 Years of School	38.5	484
University/College	32.8	412
Other	1.1	14
Occupation		
Education/Study	39.8	499
Full-time	30.9	388
Part-time	23.3	292
Unemployed	5.5	69
Parents/Parental leave	5.1	64
Unable to work	2.0	25
Other	7.3	91
Marital Status		
Single	51.7	649
In a relationship	31.4	395
Married	11.7	147
Separated/Divorced	4.8	60
Widowed	0.4	5

*Note. N* = 1,256. Multiple selections were possible for the variable occupation.

### BDD Symptoms

Although this study did not include clinical interviews, we carefully assessed BDD using the BDD-5 (Möllmann & Buhlmann, 2024), a well-developed self-report questionnaire based on DSM-5 criteria, consisting of six dichotomous items (agree/disagree). It should be noted though that while this allows for an approximation of diagnostic criteria, it does not replace a formal clinical diagnosis. The severity of BDD symptoms within the past week was assessed using the Body Dysmorphic Symptoms Questionnaire (Fragebogen körperdysmorpher Symptome: FKS) developed by Buhlmann et al. (2009). It comprises two subscales: Item 1 and Items 4-15, which queries the extent of BDD symptoms and BDD-related dysfunctional behaviors, and Items 16-18, which includes items regarding aesthetic surgeries and suicidality. Items 2 and 3 do not contribute to the total score, and were therefore not considered in further analyses, as Item 2 assesses body areas relevant to the symptoms and Item 3 serves to differentiate BDD from EDs (Buhlmann et al., 2009). The remaining items refer to symptoms within the past week and are rated on a 5-point Likert scale from 0 (not at all/never/don’t think about it at all) to 4 (very much/more than 5 times/more than 8 hours per day).^1^1Furthermore, to assess BDD symptoms in our sample, Items FKS 17 and 18, which measure suicidality, were included in the total score. For the mediation analyses, however, these items were excluded to avoid confounding effects on the dependent variable Suicidality. Similarly, to assess depressive symptoms, PHQ Item 9, which measures suicidality, was excluded in the mediation analyses. A maximum score of 64 points can be achieved, with a cut-off score of 23 points for a positive screening for BDD recently established by Meier et al. (2025). The FKS demonstrates good validity (sensitivity = .87 and specificity = .93; Buhlmann et al., 2009). The internal consistency of these items in this study was α = .80.

### Depression

The Patient Health Questionnaire (PHQ-9; Löwe et al., 2002; Spitzer et al., 1999) measures depressive symptoms over the past two weeks. The scale consists of nine items that assess depressive symptoms over the past two weeks on a 4-point Likert scale (0 = not at all, 1 = several days, 2 = more than half the days, 3 = nearly every day). The PHQ-9 serves as a screening tool for MDD and is based on the criteria for a major depressive episode according to DSM-5 (APA, 2013; sensitivity = .95 and specificity = .86; Gräfe et al., 2004). The criteria for MDD are considered met if at least five of the Items 1-9, including either Item 1 or 2, are rated as occurring more than half of the days. In the present study, only Items 1-8 of the PHQ-9 were used to measure depressive symptoms^1^. Internal consistency of the PHQ-9 in the current sample was α = .86.

### Suicidality

Suicidal thoughts and actions were assessed using FKS Items 17 ("Have you ever thought about ending your life because you find parts of your physical appearance so ugly?"), and 18 ("Have you ever attempted suicide because you find parts of your physical appearance so ugly?"), as well as PHQ-9 Item 9 ("How often have you been bothered by the following problems over the past two weeks: Thoughts that you would be better off dead or of hurting yourself?"; (hereinafter referred to as FKS_17, FKS_18, and PHQ_9).

### Statistical Analyses

The data was analyzed using IBM SPSS Statistics (Version 29.0; IBM Corporation, 2022) for descriptive data analyses, and MPlus, version 8.7 (Muthén & Muthén, 2017) for factor analyses and structural equation modeling (SEM). These analyses were conducted using Item Response Theory (IRT) models for categorial items with a robust weighted least squares mean and variance (WLSMV) adjusted estimator (Brown, 2006). To test the factorial structure of the FKS and the PHQ-9 after extracting the items measuring suicidality, exploratory factor analyses (EFA) were conducted. To determine the number of factors, the Kaiser criterion (Guttman, 1954; Kaiser & Dickmann, 1959), and the interpretability of factor structure were considered. The model quality was assessed using the Root Mean Square Error of Approximation (RMSEA; Steiger, 1980), Comparative Fit Index (CFI; Bentler, 1990) and Tucker-Lewis Index (TLI; Tucker & Lewis, 1973). A RMSEA < .05 indicates a good model fit, < .08 an acceptable model fit, and > .10 a poor model fit (Browne & Cudeck, 1992). For CFI and TLI, acceptable model fit criteria were set as ≥ .95 (Hu & Bentler, 1999). Confirmatory factor analyses (CFA) were subsequently estimated for scale adjustment. First, items with factor-loading < .35 were excluded. Then, modification indices (MI) were inspected to detect model misspecifications (starting with the MI indicating the highest change in Chi-square-statistic). Suggested residual correlations were stepwise freely estimated only if they could be interpreted meaningfully in terms of content. Another CFA was performed to examine whether the three extracted items asking for suicidality (FKS_17, FKS_18, and PHQ_9) could also be represent as latent construct, considering the variable suicidality in mediation analyses both as manifest items, and as a latent construct.

### Mediation Analyses

Four structural equation models (SEMs; Models 1a, 2a, 3a, 4a) were estimated (Geiser, 2011; Haider et al., 2023) to test a possible mediation between BDD symptoms and suicidality through depressive symptoms. Suicidality was operationalized differently: By using the manifest items FKS_17 (Model 1a with FKS_17), FKS_18 (Model 2a with FKS_18), and PHQ_9 Model 3a with PHQ_9), and by using the latent factor suicidality, represented by the three items (Model 4a with SUI).

In a second step, all four models were re-estimated including gender and age as covariates to address potential confounding influences (Models 1b, 2b, 3b, 4b). Direct, indirect, and total effects were computed using the WLSMV estimation statistic via the Sobel test (Kleinke et al., 2017). Standardized effect sizes and model quality measures were reported. Bias-corrected bootstrap method with *m* = 10000 were employed to calculate confidence intervals for effects without assuming normal distribution (Kleinke et al., 2017; MacKinnon, 2012). A significance level of α = .05 was set for all analyses.

## Results

### Sample Characteristics

The average sum score of the FKS was *M* = 37.3 (*SD* = 8.7). Almost all participants achieved the cut-off value of 23 (94.7%, *n* = 1,189). The mean sum score of the PHQ-9 was *M* = 13.4 (*SD* = 6.2). Almost half (49.8%, *n* = 625) of the sample met the criteria for a current MDD according to the PHQ-9. Two thirds of the participants (67.0%, *n* = 842) reported appearance-related suicidal thoughts (FKS_17). Of these, 23.0% had strong suicidal thoughts, and 17.0% had very strong suicidal thoughts. In our sample, general suicidal thoughts occurred in 59.6% (*n* = 748) of the people (PHQ_9). Of these people, 15.2% suffered from suicidal thoughts on more than half of the days, and 17.1% almost every day. Every twelfth person (8.5%, *n* = 107) stated that they had attempted suicide in the past at least once because of their appearance (FKS_18; see Table 2 for the response frequencies of the items on suicidality).

**Table 2:** Frequencies of Suicidality Items

Item and Response	%	*n*
FKS_17 Have you ever thought about taking your own life because you find parts of your physical appearance so ugly?		
Not at all	33.0	414
A little	24.4	307
Moderate	15.8	198
Strong	15.4	194
Very strong	11.4	143
FKS_18 Have you ever attempted to take your own life because you find parts of your physical appearance so ugly?		
Never	91.5	1149
Once	6.1	76
Twice	1.2	15
3 to 5 times	0.6	8
More than 5 times	0.6	8
PHQ_9 Over the last 2 weeks, how often have you been bothered by: Thoughts that you would be better off dead or of hurting yourself in some way?		
Not at all	40.4	508
On some days	40.3	506
On more than half of the days	9.1	114
Almost every day	10.2	128

*Note*. *N* = 1,256. FKS_17 = Item 17 of the FKS = Body Dysmporphic Symptoms Questionnaire; FKS_18 = Item 18 of the FKS = Body Dysmporphic Symptoms Questionnaire; PHQ_9 = Item 9 of the Patient Health Questionnaire.

### Measurement Models

#### BDD Symptoms

The exploratory factor analysis of the FKS items revealed four eigenvalues greater than one (Guttman, 1954; Kaiser & Dickmann, 1959). The two-factor solution did not exhibit a clear loading structure. Items FKS 11, FKS 13, FKS 15, and FKS 16 could not be assigned to any factor. All items showed sufficient positive loadings on both factors, and the difference in loadings of these items between the two factors was less than .20. Only Items FKS 9 and FKS 14, which pertained to verbal expressions of appearance concerns, could be clearly assigned to the second factor. Thus, a meaningful interpretation of the two-factor solution was not possible. The interpretability of a three- and four-factor solution was also not possible. Subsequently, a single-factor model was selected, but it demonstrated poor fit indices (χ^2^(77, *N* = 1,256) = 2,041.25, *p* < .001; RMSEA = .14; CFI = .85; TLI = .83; Hu & Bentler, 1999). From this model two items were excluded due to low factor loadings (FKS 12, FKS 16). Based on modification indices three residual correlations were freely estimated (FKS 9 with FKS 14; FKS 6 with FKS 7; FKS 8 with FKS 15), resulting in a final model with satisfactory fit indices (χ^2^(51, *N* = 1,256) = 512.16, *p* < .001; RMSEA = .09; CFI = .96; TLI = .95). Detailed tables listing the loadings for each individual questionnaire item can be found in the Supplementary Materials, Appendix 1.

#### Depression

An exploratory factor analysis (EFA) was conducted for the PHQ-items. The Kaiser criterion indicated a single-factor solution, as only the first factor had an eigenvalue > 1 (cf. Cattell, 1966; Guttman, 1954). Therefore, a multifactorial solution was rejected. All item-to-factor-loadings were sufficiently high (> .50; cf. Moosbrugger & Kelava, 2012). Two residual correlations were freely estimated (PHQ 1 with PHQ 2; PHQ 3 with PHQ 4). For this model, fit indices indicated a good model fit (χ^2^(19, *N* = 1,256) = 79.92, *p* < .001; RMSEA = .05; CFI = .99; TLI = .99). Comprehensive tables with item-level loadings are provided in the Supplementary Materials, Appendix 2.

#### Suicidality

Since the confirmatory factor analysis with the Items FKS_17, FKS_18, and PHQ_9 was a saturated model with zero degrees of freedom, the model had a perfect fit (χ^2^(0, *N* = 1,256) = .00, *p* < .001; RMSEA = .00; CFI = 1.00; TLI = 1.00; cf. Geiser, 2011). The exact factor loadings for each individual questionnaire item can be found in the Supplementary Materials, Appendix 3.

### Structural Equation Models

**Figure 1:**
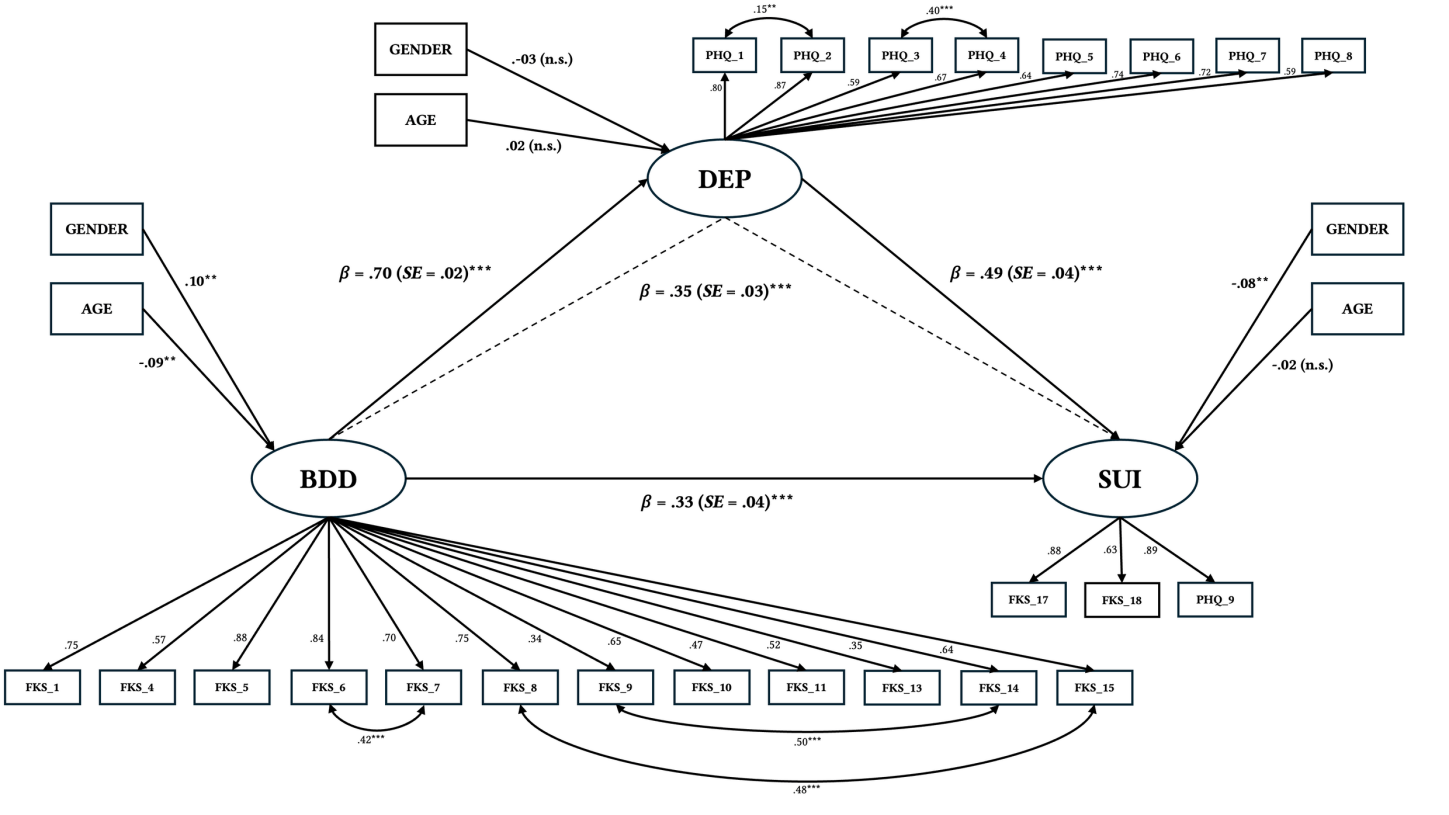
Structural Equation Model (4b): Mediation of Depressive Symptoms in the BDD-Suicidality Link *Note.* All paths in the structural equation model were adjusted for age and gender. Statistics are standardized regression coefficients (β) with standard errors (*SE*) in parenthesis, and standardized factor loadings from structural equation. Solid lines represent direct effects; dashed lines indicate the indirect effect; standardized residual correlations are depicted as solid double-headed arrows. All factor loadings were statistically significant (*p* < .001). BDD = BDD symptoms; DEP = depressive symptoms; SUI = suicidality; FKS_17 = Item 17 of the Body Dysmorphic Symptoms Questionnaire; FKS_18 = Item 18 of the Body Dysmorphic Symptoms Questionnaire; PHQ_9 = Item 9 of the Patient Health Questionnaire; n.s. = not significant. **p < .01. ***p < .001.

The computed models are reported below (see Table 3 and in the Supplementary Materials, Appendix 4). The most comprehensive model, including the latent variable suicidality with gender and age as covariates, is shown in Figure 1.

#### Model 1a With FKS_17

The model fit of Model 1a was acceptable (χ^2^(182, *N* = 1,256) = 1,290.26, *p* < .001; RMSEA = .07; CFI = .95; TLI = .94). The relationship between BDD symptoms and appearance-related suicidal thoughts (FKS_17) could be partially explained by an indirect effect via depression (β = .20, *p* < .001). The direct effect of BDD symptoms on Item FKS_17 was also significant (β = .42, *p* < .001; see Table 3 for more details).

#### Model 2a With FKS_18

The fit indices indicated acceptable model fit (χ^2^(182, *N* = 1,256) = 1,189.75, *p* < .001; RMSEA = .07; CFI = .96; TLI = .95). The indirect effect via depression explained part of the association between BDD symptoms and appearance-related suicide attempts (FKS_18), (β = .20, *p* < .01). In addition, there was a direct effect between BDD symptoms and the Item FKS_18 (β *=* .28, *p* < .01; see Table 3).

**Table 3:** Structural Equation Models: Mediation of Depressive Symptoms in the BDD–Suicidality Link

Model	Effect	Predictor → Outcome	Effect size	*SE*	95% CI	*p*
1a	Total effect	BDD → FKS_17	.62***	.02		
	Direct effects	BDD → DEP	.70***	.02	[.65, .75]	< .001
		DEP → FKS_17	.28***	.04	[.17, .39]	< .001
		BDD → FKS_17	.42***	.04	[.31, .53]	< .001
	Indirect effect	BDD → DEP → FKS_17	.20***	.03	[.12, .28]	< .001
2a	Total effect	BDD → FKS_18	.44***	.04		
	Direct effects	BDD → DEP	.70***	.02	[.65, .75]	< .001
		DEP → FKS_18	.28**	.09	[.05, .51]	.002
		BDD → FKS_18	.25**	.09	[.03, .47]	.004
	Indirect effect	BDD → DEP → FKS_18	.20**	.06	[.04, .36]	.002
3a	Total effect	BDD → PHQ_9	.54***	.03		
	Direct effects	BDD → DEP	.70***	.02	[.65, .75]	< .001
		DEP → PHQ_9	.62***	.04	[.52, .72]	< .001
		BDD → PHQ_9	.10*	.04	[-.01, .22]	.019
	Indirect effect	BDD → DEP → PHQ_9	.43*	.03	[.35, .52]	< .001
4a	Total effect	BDD → SUI	.66***	.02		
	Direct effects	BDD → DEP	.70***	.02	[.65, .75]	< .001
		DEP → SUI	.50***	.04	[.39, .60]	< .001
		BDD → SUI	.31***	.04	[.21, .42]	< .001
	Indirect effect	BDD → DEP → SUI	.35***	.04	[.27, .43]	< .001

*Note.* Structural equation models tested depressive symptoms as a mediator of the association between BDD symptoms and suicidality. Suicidality operationalised as: (1a) appearance-related suicidal thoughts (FKS_17); (2a) appearance-related suicide attempts (FKS_18); (3a) general suicidal thoughts (PHQ_9); (4a) latent factor suicidality (FKS_17, FKS_18, PHQ_9). Standardized effects with bias-corrected bootstrap 95% CIs are reported. CI = confidence interval; BDD = BDD symptoms; DEP = depressive symptoms; SUI = suicidality; FKS_17 = Item 17 of the Body Dysmorphic Symptoms Questionnaire; FKS_18 = Item 18 of the Body Dysmorphic Symptoms Questionnaire; PHQ_9 = Item 9 of the Patient Health Questionnaire.

*p < .05. **p < .01. ***p < .001.

#### Model 3a With PHQ_9

The fit of the model was acceptable (χ^2^(182, *N* = 1,256) = 1,248.04, *p* < .001; RMSEA = .07; CFI = .95; TLI = .95). There was both a direct effect (β = .10, *p* < .05) and an indirect effect (β = .43, *p* < .001) via depression between BDD symptoms and general suicidal thoughts (PHQ_9; see Table 3).

#### Model 4a With SUI

The model fit was acceptable (χ^2^(222, *N* = 1,256) = 1,418.53, *p* < .001; RMSEA = .07; CFI = .95; TLI = .95). The relationship between BDD symptoms and the latent variable suicidality could be partially explained by an indirect effect via depression (β *=* .35, *p* < .001). There was also a direct correlation between BDD symptoms and suicidality (β *=* .32, *p* < .001; see Table 3).

#### Model 1b, 2b, 3b, 4b Including Gender and Age as Covariates

All effects described in the previous models remained significant even when controlling for gender and age. See in the Supplementary Materials, Appendix 4 for specific total, direct, indirect effects, and bootstrapping-confidence intervals for Model 1b, 2b, 3b, 4b and Figure 1 for Model 4b specifically.

## Discussion

The aim of the present study was to explore the relationships between BDD symptoms, depression, and suicidality among an online sample of individuals with BDD (based on self-report measures). Specifically, participants reported high levels of BDD symptoms, even slightly exceeding those found in clinical samples diagnosed with BDD (e.g., Buhlmann et al., 2011; Hübner et al., 2016), with skin, nose, and hair being the most frequently mentioned areas of concern, which is very similar to those areas found in other clinical samples (e.g., Phillips et al., 2005a). Almost half of our sample (49.8%) met the criteria for MDD (based on self-report), which was slightly lower than in Gunstad and Phillips (2003; 61% phenomenological study, 54% treatment study). Moreover, two thirds (67%) of the participants reported appearance-related suicidal thoughts, which is comparable with the findings of Angelakis et al. (2016; 19.1 to 69.7%). 59.6% of our sample reported experiencing general suicidal thoughts within the past two weeks. This rate is considerably higher than the average prevalence of current suicidal thoughts (37.2%) found in clinical BDD populations, as reported in a meta-analysis by Pellegrini et al. (2021). Moreover, consistent with previous research (e.g., Angelakis et al., 2016) 8.5% of participants in this study disclosed a previous suicide attempt specifically attributed to BDD symptoms. The elevated frequency of suicide attempts in BDD is particularly concerning, as past suicide attempts constitute a significant risk factor for completed suicide (Bostwick et al., 2016).

### Depressive Symptoms as a Potential Mediator in the Relationship Between BDD and Suicidality

The hypothesis that depression mediates the relationship between BDD symptoms and suicidality was supported by our data. Specifically, BDD symptoms were consistently positively associated with depressive symptoms across all models (β = .70), aligning with prior research linking BDD severity to increased depression (Phillips et al., 2007a) as well as with epidemiological evidence documenting the high prevalence of MDD among individuals with BDD (Gunstad & Phillips, 2003; Phillips et al., 2005a; 2007b). In turn, depressive symptoms showed a significant direct effect on suicidality, consistent with previous findings indicating that depression is a key correlate of suicidal ideation and behavior (Brown et al., 2000; Snorrason et al., 2019) and that there are elevated rates of suicidality in MDD (Cai et al., 2021; Dong et al., 2018). The association between depression and suicidality varied depending on the suicidality measure (Model 1a with FKS_17, Model 2a with FKS_18, Model 3a with PHQ_9, Model 4a with SUI), with the strongest effect observed for general suicidal thoughts (Model 3a, β = .62), comparable to the finding by Shaw et al. (2016). Regarding the mediation hypothesis, small indirect effects via depression were found between BDD symptoms and appearance-related suicidal thoughts (Model 1a, β = .20) and between BDD symptoms and appearance-related suicide attempts (Model 2a, β = .20). Interestingly, a moderate indirect effect via depression was found between BDD symptoms and general suicidal thoughts (Model 3a, β = .43). The finding of small indirect effects for appearance-related suicidality and a moderate indirect effect for general suicidality may point to the potential importance of distinguishing between appearance-related and general suicidal thoughts in individuals with BDD. However, it should be noted that a potential confounding factor is that the items used as dependent variables (FKS_17, FKS_18, PHQ_9) were extracted from questionnaires (FKS, PHQ-9) that also contributed to the independent variables or mediators in the models. This could potentially inflate effects between the affected constructs due to methodological artifacts. To address this, we constructed a composite suicidality variable (SUI; see Models 4a/4b in Figure 1, Table 3, and in the Supplementary Materials, Appendix 4) by combining the indicators of both questionnaires into a latent construct to reduces potential bias from item-level overlap by capturing the shared variance among the three indicators. This model revealed a moderate indirect effect of BDD symptoms on the latent variable suicidality (Model 4a, β = .35).

In sum, our results are partly consistent with prior research indicating that depressive symptoms partially (but not fully) mediate the association between BDD and suicidality, as reported by Shaw et al. (2016). However, unlike Shaw et al. (2016), who recruited participants based on self-reported appearance-related concerns without formally establishing a clinical diagnosis of BDD and investigated constructs theoretically linked to suicidal desire rather than suicidality directly, our study included individuals based on a validated self-report screening tool assessing established diagnostic criteria for BDD and employed latent measures of suicidality.

### Direct Associations Between BDD Symptoms and Suicidality

The second hypothesis was statistically supported, showing a direct association of BDD symptoms on suicidality across all eight models, consistent with previous research (Krebs et al., 2022; Phillips et al., 2005c; Snorrason et al., 2019). A strength of our study was that we estimated the magnitude of the total effects, as well as the direct and indirect effects. The direct association between BDD symptoms on appearance-related suicidal thoughts was stronger (Model 1a, β = .42) than the association with appearance-related suicide attempts (Model 2a, β = .28). This discrepancy might reflect the general difficulty in predicting suicide attempts compared to suicidal thoughts (Glenn & Nock, 2014; Ribeiro et al., 2019). Remarkably, a small but significant direct association was found between BDD symptoms and general suicidal thoughts (Model 3a, β = .10), diverging from findings by Shaw et al. (2016), who reported a full mediation via depression. However, Shaw et al. (2016) only examined the relationship between BDD symptoms and perceived burdensomeness and thwarted belongingness. These constructs may be more closely linked to depression than direct assessments of suicidality, potentially resulting in stronger predictability by depression. Overall, these findings suggest that depressive symptoms may not entirely account for suicidality in BDD.

### Influence of Gender and Age on BDD Symptoms and Suicidality

Across all adjusted models (Models 1b–4b), adding gender and age as covariates did not substantially alter the size or significance of the direct and indirect effects (see Supplementary Materials, Appendix 4 and Figure 1).

### Clinical Implications

BDD is characterized by perceived defects of one’s own physical appearance (Veale et al., 2016), which is often accompanied by rigid and sometimes delusional appearance beliefs. Affected individuals often feel misunderstood because they perceive something in a way that others do not (Brito et al., 2014). This may lead to increased hopelessness or increased risk of suicidality. Based on our findings, suicidality should be assessed in patients with BDD, regardless of comorbid depressive symptoms. In terms of clinical interventions, it is important to address BDD-typical risk factors (e.g., social withdrawal due to feelings of negative evaluation by others, not belonging to a social group or being a burden to others) into the treatment. General depression-focused interventions might not sufficiently capture the risk factors associated with suicidality in BDD.

### Limitations and Directions for Future Research

This study has several limitations. First, BDD symptoms were assessed using self-report measures. This limits diagnostic accuracy and raises the risk of overlap with related disorders such as social anxiety disorder or obsessive-compulsive disorder (Schulte et al., 2020). Thus, future studies should incorporate structured clinical interviews to ensure more accurate diagnostic BDD assessment, including the assessment of possible comorbidities. It should be noted though that our sample had very comparable levels of BDD symptom severity and suicidality to those found in diagnosed clinical samples, which supports the idea that online studies can be a useful tool to particularly reach those individuals with BDD who might otherwise not be able to be reached (e.g., due to treatment barriers, being housebound). Second, the operationalization of suicidality was limited in parts of the study. In Models 1a/b, 2a/b, and 3a/b, suicidality was assessed using single items (FKS_17, FKS_18, PHQ_9), which may have led to skewed response distributions, potentially affecting the reliability and sensitivity of the measure. Moreover, single-item measurement is generally considered less reliable and more vulnerable to method effects, as items from the same questionnaire may correlate more strongly with each other than with conceptually similar items from different sources (Nunnally & Bernstein, 1994). However, in the SUI Model (4a/b), suicidality was modeled as a latent variable based on three indicators (FKS_17, FKS_18, PHQ_9), which partially reduces these limitations by allowing for more robust construct modeling. To further improve measurement precision, future studies should implement validated multi-item scales such as the Suicidal Ideation and Behavior Scale (SSEV; Teismann et al., 2021), which offer a more differentiated assessment of suicidal thoughts and behaviors. Third, the cross-sectional design of our study precludes conclusions about temporal or causal relations. Longitudinal research is needed to determine the directionality and potential bidirectionality of the relationships between BDD symptoms, depression, and suicidality. Lastly, the sample was predominantly female, limiting generalizability to male or gender-diverse individuals. To increase external validity, future studies should aim for more gender balanced samples.

Nonetheless, the present study includes a substantially larger sample size compared to earlier research (e.g., Phillips et al., 2005c, *N* = 200; Shaw et al., 2016, *N* = 235 participants), which enhances the statistical power and generalizability of our findings.

With respect to future research, additional psychosocial risk factors such as self-esteem (Kuck et al., 2021; Soto-Sanz et al., 2019), bullying and childhood trauma (e.g. abuse and neglect; Rück et al., 2024), or reduced quality of life (de Freitas Melo et al., 2022; Phillips et al., 2005b) should be considered as further explanatory variables in future models to better understand the complex mechanisms underlying suicidality in BDD.

### Conclusion

The study underscores the elevated risk of suicidality among individuals with BDD, emphasizing the importance for clinical professionals to carefully assess suicidality when encountering signs of BDD. While depressive symptoms partially mediate the relationship between BDD symptoms and suicidality, the study suggests that BDD symptoms are associated with increased suicidality irrespective of depressive symptoms. This highlights the need for simultaneous treatment of both BDD-related symptoms and suicidality (Dozois & Rnic, 2015; Wilhelm et al., 2012). However, due to the cross-sectional design, causal conclusions cannot be drawn. Future research is needed to examine the association of BDD and suicidality using different methods and designs including clinician-administered assessment methods and longitudinal designs.

## Supplementary Materials

The Supplementary Materials contain the following items:

- Research data and analysis code (Vogel, 2025S)
- Online appendices (Vogel et al., 2026S):
  - *Appendix 1* presents the results of an exploratory factor analysis for FKS Items 1 and 4–16 (One-Factor Model).
  - *Appendix 2* presents the results of an exploratory factor analysis for PHQ-9 Items 1–8 (One-Factor Model).
  - *Appendix 3* presents the results of an exploratory factor analysis for the latent factor of suicidality.
  - *Appendix 4* presents the structural equation models testing the mediation of depressive symptoms in the association between BDD symptoms and suicidality.

VogelH.
 (2025S). The relationship between body dysmorphic symptoms, depressive symptoms, and suicidality: A mediation analysis
[Research data and analysis code]. PsychOpen. 10.17605/OSF.IO/AB8X4
PMC1295530541783468

VogelH.
BarenbrüggeJ.
JenischJ.
SchulteJ.
BuhlmannU.
 (2026S). Supplementary materials to "The relationship between body dysmorphic symptoms, depressive symptoms and suicidality – A mediation analysis"
[Online appendices]. PsychOpen. 10.23668/psycharchives.21629
PMC1295530541783468

## Data Availability

To support transparency and openness in science, data and analysis code is provided on the Open Science Framework (OSF; https://doi.org/10.17605/OSF.IO/AB8X4).

## Preregistration

There is no preregistration for this study or analysis plan.

## Reporting Guidelines

This article is written according to the JARS-QUANT guidelines.
